# (4-(Adamantan-1-yl)-1-(isopropyl)-1*H*-imidazol-2-yl)methanol

**DOI:** 10.3390/M1566

**Published:** 2023-01-23

**Authors:** Ryan B. Gaynor, Baylee N. McIntyre, Sidney E. Creutz

**Affiliations:** Department of Chemistry, Mississippi State University, Mississippi State, MS 39762, USA

**Keywords:** imidazoles, ligand precursors, bioinspired

## Abstract

(4-(Adamantan-1-yl)-1-(isopropyl)-1*H*-imidazol-2-yl)methanol was prepared through a five-step process starting from commercially available 1-acetyladamantane. Each step proceeded in moderate-to-excellent yields and the overall yield across five steps was 28%. The compound was identified and characterized by ^1^H and ^13^C{^1^H} NMR, high-resolution mass spectroscopy, and elemental analysis. This compound and its derivatives have the potential to be used as precursors to the synthesis of biomimetic chelating ligands.

## 1. Introduction

The amino acid histidine plays a key role in bioinorganic chemistry due to the propensity of the imidazole moiety to act as a donor ligand to metal atoms. Prominent examples include hemoglobin, where the iron porphyrin oxygen carrier bears an axial histidine donor; methane monooxygenase, whose active site contains two iron centers both coordinated by a histidine donor; a large group of zinc proteinases containing a tripodal tris (histidine) zinc binding site; and the protein calprotectin, which includes a hexahistidine metal binding site putatively used to sequester metal ions as an immune defense strategy [1–5]. Because of this, imidazole groups are commonly incorporated into biomimetic model complexes designed to mimic the structure and/or function of metalloproteins [5–8]. The design and optimization of chelating ligands for this purpose requires the ability to access functionalized imidazole-containing synthons containing substituents with different steric and electronic properties. Here, we report the synthesis of the new molecule (4-(adamantan-1-yl)-1-(isopropyl)-1H-imidazol-2-yl)methanol (**5**). This molecule, as well as its precursors **3** and **4**, are potential starting materials for further elaboration into new chelating ligands. The bulky adamantyl group would be expected to provide a substantial steric shielding of a coordinated metal center—this steric shielding could promote the study of reaction pathways and reactive intermediates.

## 2. Results and Discussion

**Scheme 1.**
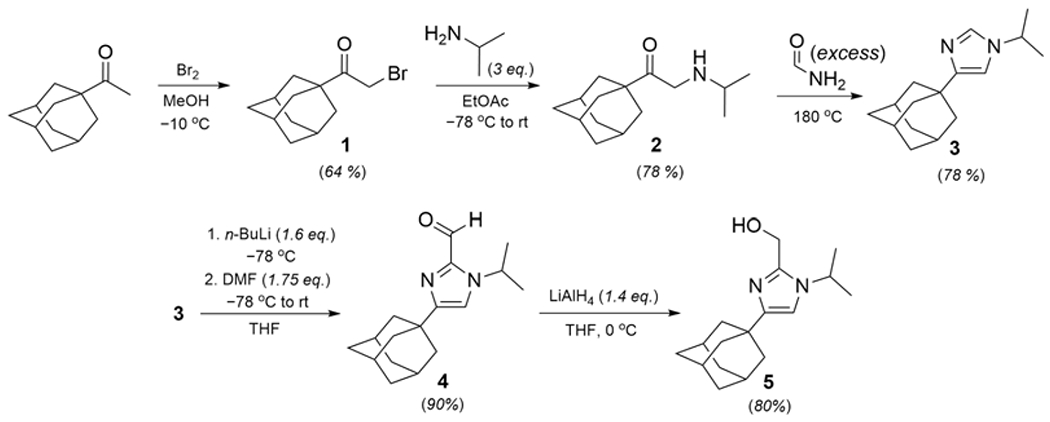
Preparation of compound **5**. DMF = dimethylformamide; THF = tetrahydrofuran.

The synthesis of **5** requires five steps from commercially available 1-acetyladamantane and is shown in Scheme 1; the NMR spectra of the product and intermediates are provided in the Supplementary Material. The *α*-brominated ketone precursor **2** has been reported but was previously synthesized by different routes [9–11]; our approach is adapted from a procedure used for the synthesis of 1-bromo-3-methyl-2-butanone and has been successfully carried out on up to a 10 g scale [12]. The next two steps (amination with isopropylamine and cyclization with formamide to give **3**) are adapted from a reported synthesis of other closely related 1,4-disubstituted imidazoles [13]. Intermediates **1** and **3** could be isolated and purified; however, neat samples of **2** decompose over the course of hours to days, and this compound should be used immediately for subsequent reactions without purification. The decomposition reaction is hypothesized to involve a self-condensation reaction to give a substituted dihydropyrazine; similar pathways have been observed for related aminoketone compounds [13].

The disubstituted imidazole **3** was further functionalized by lithiation with *n*-butyllithium followed by reaction with dimethylformamide as a C_1_ source to introduce a formyl group in the 2-position, giving **4**, followed by reduction with lithium aluminum hydride to give the title compound **5**. Compound **5** has been fully characterized by ^1^H NMR, ^13^C{^1^H} NMR, high-resolution mass spectrometry (HRMS), and CHN combustion analysis; all data are consistent with the proposed structure.

## 3. Materials and Methods

### 3.1. General Synthesis

*N*-isopropylimidazole was purchased from Accela and 1-acetyladamatane was purchased from Thermo Scientific. All other starting materials, reagents, and solvents were purchased from standard chemical vendors and used without further purification unless described otherwise below. All reactions were carried out under an atmosphere of anhydrous nitrogen or argon gas; workups and characterization were carried out under air.

HRMS were performed on a Bruker micrOTOF-QII using electrospray ionization (ESI). Electron impact mass spectrometry (EI-MS) was performed on a Shimadzu GCMS-QP2010 Ultra using compounds introduced as methylene chloride solutions. Elemental analysis was carried out using an Elementar UNICUBE analyzer, which was standardized using sulfanilamide. ^1^H NMR and ^13^C{^1^H} NMR spectra were measured on a Bruker Avance NMR system equipped with a 500 MHz or 300 MHz superconducting magnet. NMR spectra were referenced internally to the residual CHCl_3_ (for ^1^H NMR, 7.26 ppm) or CDCl_3_ signal (for ^13^C NMR, 77.16 ppm).

### 3.2. Synthesis of 1-(Adamantan-1-yl)-2-bromoethanone (1)

The synthesis was adapted from a procedure previously reported for the preparation of the related compound 1-bromo-3-methyl-2-butanone [12]. To a round-bottomed flask with a stir bar, 10.0 g (0.0561 mol) of 1-acetyladamantane was added along with dry methanol and was placed under inert gas on a Schlenk line. The flask was placed in an ice/salt bath and cooled to −10 °C. To the stirred solution, 2.88 mL (0.0561 mol, 1 eq.) of bromine was rapidly injected with a syringe. This was left to stir for 2 h while ensuring that the reaction temperature never exceeded 10 °C. After this two-hour period of cold stirring, 30 mL of deionized water were added and the flask was removed from the ice bath and left to stir overnight at room temperature. Potassium carbonate was added until the solution became colorless, followed by 100 mL of saturated aqueous sodium chloride. The product was extracted three times with 75 mL diethyl ether and the organic layers were combined and dried with anhydrous sodium sulfate and filtered, followed by removal of the solvent by rotary evaporation. Cold methanol was added to the resulting oily residue, causing a white solid product to precipitate. The precipitate was collected via filtration and washed with cold methanol, and dried, affording a white crystalline product (9.2 g, 63.7%). ^1^H NMR (500 MHz, CDCl_3_, 25 °C): δ 4.15 (s, 2H), 2.07 (m, 3H), 1.88 (d, *J* = 5 Hz, 6H), 1.76 (m, 3H), 1.70 (m, 3H) ppm; ^13^C{^1^H} NMR (126 Hz, CDCl_3_, 25 °C): δ 205.6, 46.6, 38.5, 36.3, 31.9, 27.8 ppm. Elemental Analysis. Calcd. For C_12_H_17_BrO: C, 56.05; H, 6.66. Found: C, 56.26; H, 6.75. Spectroscopic data are consistent with previous reports [10].

### 3.3. Synthesis of 1-(Adamantan-1-yl)-2-(isopropylamino)ethanone (2)

This synthesis is adapted from previously reported preparations of related 1-alkyl-2-(isopropylamino)ethanone compounds [13,14]. To a three-neck round-bottomed flask with a stir bar, 2.8 mL of isopropylamine (0.0326 mol, 3 eq.) was added with 50 mL of ethyl acetate. This was cooled to −78 °C with a dry ice/isopropanol bath and placed under an inert atmosphere on a Schlenk line. A pressure equalizing addition funnel was attached to the round-bottomed flask and 2.8 g of 1-(adamantan-1-yl)-2-bromoethanone (**1**, 0.0109 mol, 1 eq.) was dissolved in about 25 mL of ethyl acetate and added to the addition funnel. This was added dropwise over 15 min, after which the flask was allowed to stir at −78 °C for 4 h, before warming to room temperature while stirring overnight. Next, 15 mL of 15% NaOH solution were added, and the resulting solution was extracted with ethyl acetate (3 × 100 mL). The organic layers were combined and washed with water and then brine. The organic layers were then dried with anhydrous magnesium sulfate and filtered, and the solvent was evaporated, revealing a yellow oil (2.00 g, 78%). Because this compound decomposes upon standing, the crude material was used immediately and without further purification for the next step. ^1^H NMR (500 MHz, CDCl_3_, 25 °C): δ 3.60 (s, 2H), 2.68 (spt, *J* = 6.2 Hz, 1H), 2.05 (m, 3H), 1.83 (d, *J* = 2.3 Hz, 6H), 1.73 (m, 6H) ppm, 1.06 (d, *J* = 6.2 Hz, 6H).

### 3.4. Synthesis of 4-(Adamantan-1-yl)-1-isopropylimidazole (3)

This process was adapted from the previously reported synthesis of the related compound 1,4-diisopropylimidazole [13,14]. To a 500 mL three-neck round-bottomed flask equipped with a stir bar, 35 mL of formamide was added. An air-cooled reflux condenser was affixed to the center neck, a thermometer was inserted in one neck, and the third neck was equipped with an addition funnel containing 4.02 g (0.0171 mol) of 1-(adamantan-1-yl)-2-(isopropylamino)ethanone (**2**) suspended in 20 mL of ethyl acetate. A gas inlet adaptor on top of the addition funnel was used to introduce an inert gas (nitrogen or argon). The top of the reflux condenser was left open to allow the inert gas flow to flush out water vapor; the gas flow rate was adjusted so that, once the reaction was begun, water vapor exhausted from the top of the condenser at a slow but steady rate. The formamide was heated to 180° C and the 1-(adamantan-1-yl)-2-(isopropylamino)ethanone solution was added dropwise over 30 min; stirring was then continued at 180 °C for 3 h before the reaction was allowed to cool to room temperature. About 70 mL of DI water was added, followed by 20 mL of 15% aqueous NaOH. The product was extracted with twice with approximately 75 mL of toluene, and the combined toluene extracts were washed with water and brine, then dried over anhydrous sodium sulfate and filtered. The toluene was evaporated, leaving a dark yellow oil which was further purified by elution through a silica column using EtOAc as the eluent (*R_f_* = 0.38) (3.25 g, 78%). ^1^H NMR (500 MHz, CDCl3, 25 °C): δ 7.43 (s, 1H), 6.60 (s, 1H), 4.25 (spt, *J* = 6.7 Hz, 1H), 2.04 (m, 3H), 1.91 (m, 6H), 1.76 (m, 6H), 1.46 (d, *J* = 6.7 Hz, 6H) ppm; ^13^C{^1^H} NMR (126 MHz, CDCl_3_, 25 °C): δ 152.9, 134.1, 109.9, 48.9, 42.5, 37.0, 28.7, 23.7 ppm. ESI-HR-MS (positive). Calcd. For [C_16_H_25_N_2_]^+^ (L + H)^+^: *m/z* 245.2012. Found: *m/z* 245.2069.

### 3.5. Synthesis of 4-(Adamantan-1-yl)-1-isopropylimidazole-2-carbaldehyde (4)

This synthesis was adapted from previously reported procedures used to access related substituted imidazole-2-carbaldehyde compounds [15,16]. Compound **3** (1.53 g, 0.00626 mol, 1 eq.) was dissolved in a Schlenk flask with 100 mL dry THF and placed under inert gas on the Schlenk line. This flask was added to a dry ice/isopropanol bath at −78 °C. *n*-Butyllithium (6.3 mL, 0.01 mol, 1.5 eq.) was added dropwise, and the flask was allowed to stir at −78 °C for 1.5 h. Anhydrous DMF (0.8 g, 0011 mol, 1.75 eq.) was then added to the stirred solution, where it was allowed to stir for 4 h at −78 °C, and then allowed to continue to stir while slowly warming to room temperature overnight. Next, 50 mL of DI water was added to the reaction flask and the mixture was transferred to a separatory funnel, where the mixture was extracted with diethyl ether (3 × 40 mL). The organic layers were combined and washed with water and then brine, before drying with anhydrous sodium sulfate. The sodium sulfate was removed by filtration and the organic layer was evaporated, revealing a yellow oil of crude product (1.17 g, 90%). The crude product can be crystallized from cold diethyl ether but was generally used without further purification for the next step. ^1^H NMR (500 MHz, CDCl_3_, 25 °C) δ 9.79 (s, 1H), 7.04 (s, 1H), 5.44 (m, *J* = 6.6 Hz, 1H), 2.08 (bs, 3H), 1.94 (d, *J* = 2.6 Hz, 6H), 1.78 (bs, 6H), 1.44 (d, *J* = 6.6 Hz, 6H) ppm. ^13^C{^1^H} NMR (126 MHz, CDCl_3_, 25 °C): δ 182.2, 155.5, 142.1, 116.4, 48.6, 42.3, 36.8, 33.9, 28.5, 23.5. Elemental Analysis. Calcd. For C_17_H_24_N_2_O: C, 74.96; H, 8.88; N, 10.28. Found: C, 75.77; H, 9.22; N, 10.28. ESI-HR-MS (positive). Calcd. For [C_16_H_24_N_2_ONa]^+^ (L+ Na)^+^: *m/z* 295.1781. Found: *m/z* 295.1842.

### 3.6. Synthesis of (4-(Adamantan-1-yl)-1-isopropylimidazol-2-yl) methanol (5)

This synthesis was adapted from a previously reported procedure used to access related substituted (imidazol-2-yl)methanol compounds [17]. Lithium aluminum hydride (0.724 g, 0.0191 mol, 1.4 eq.) was dissolved in 50 mL dry THF in a 250 mL round-bottomed flask under inert gas on a Schlenk line and cooled in an ice/water bath. Compound **4** (3.71 g, 0.0136 mol, 1 eq.) was separately dissolved in roughly 10 mL dry THF and added dropwise to the stirred lithium aluminum hydride solution under an inert gas. This was allowed to stir for 5 min, then 0.7 mL DI H_2_O, 0.7 mL 15% NaOH, and 2.2 mL DI H_2_O were added consecutively by syringe, slowly and dropwise (caution: significant gas evolution). The reaction was then allowed to stir for 2.5 h. Anhydrous magnesium sulfate was added to the mixture, and then the solution was filtered. The MgSO_4_ residue was washed two more times with 25 mL THF and the filtrates were combined. The THF was removed via evaporation to give an oily residue, which was triturated with hexane, crashing out a white powder. The powder was collected by filtration on a glass frit, washed with hexane, and further dried in vacuo to give the final product (0.84 g, 80%). ^1^H NMR (500 MHz, CDCl_3_, 25 °C): δ 6.57 (s, 1H), 4.69 (s, 2H), 4.58 (bs, 1H), 4.55 (spt, *J* = 6.6 Hz, 1H), 2.00 (bs, 3H), 1.81 (d, *J* = 2.4 Hz, 6H), 1.73 (s, 6H), 1.42 (d, *J* = 6.6 Hz, 6H) ppm. ^13^C{^1^H} (126 MHz, CDCl_3_, 25 °C): 151.0, 145.3, 109.1, 56.7, 47.2, 42.3. 36.9, 33.4, 28.6, 23.7 ppm. Elemental Analysis. Calcd. For C_17_H_26_N_2_O: C, 74.41; H, 9.55; N, 10.21. Found: C, 74.03, H, 9.51; N, 10.33. ESI-HR-MS (positive). Calcd. For [C_17_H_27_N_2_O]^+^ (L + H)^+^: *m/z* 275.2118. Found: *m/z* 275.2142.

## 4. Conclusions

Starting from 1-acetyladamantane, (4-(adamantan-1-yl)-1-(isopropyl)-1H-imidazol-2-yl)methanol (**5**) was synthesized in a five-step procedure. Several synthetic intermediates were also characterized, including the adamantyl-substituted imidazole derivates **3** and **4**. Compounds **3**–**5** may be useful as precursors to bulky chelating ligands for biomimetic metal complexes.

## Supplementary Material

Supplementary Data

## Data Availability

The data presented in this paper can be found within the Supplementary Materials.
