# Supplementary material for: (4-(Adamantan-1-yl)-1-(isopropyl)-1H-imidazol-2-yl)methanol
Source: Molbank. Author manuscript; Available in PMC 2023 Nov 17. (PMC10655578; doi:10.3390/M1566)
Supplement: Supplementary Data [file NIHMS1940013-supplement-Supplementary_Data.pdf]

Supplementary Materials for:

(4-(Adamantan-1-yl)-1-(isopropyl)-1H-imidazol-2-yl)methanol

Ryan B. Gaynor, Baylee N. McIntyre, and Sidney E. Creutz*

Department of Chemistry, Mississippi State University, Mississippi State, MS 39762

**screutz@chemisty.msstate.edu*

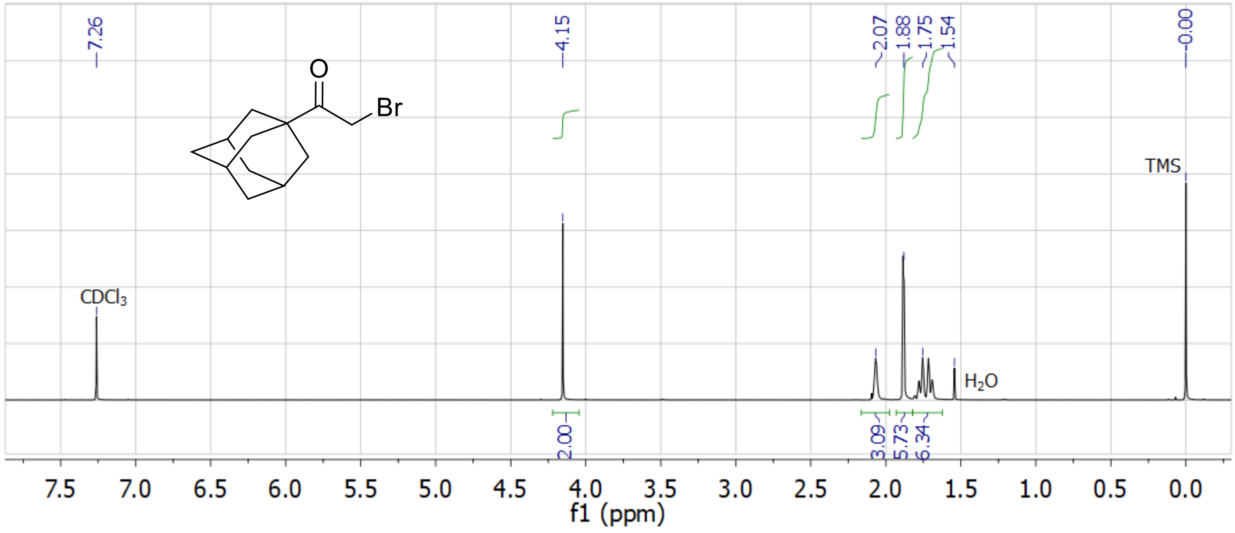

Figure S1. ^{1}H NMR of 1-(adamantan-1-yl)-2-bromoethanone (**1**). ($CDCl_3$, 500 MHz).

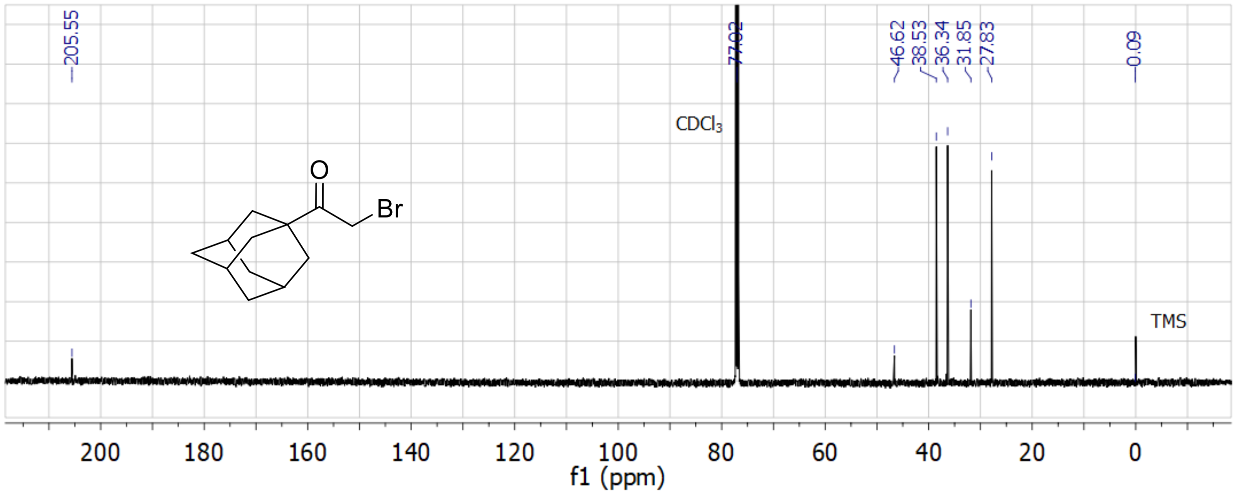

Figure S2. $^{13}C\{^1H\}$ NMR of 1-(adamantan-1-yl)-2-bromoethanone (**1**). ($CDCl_3$, 126 MHz).

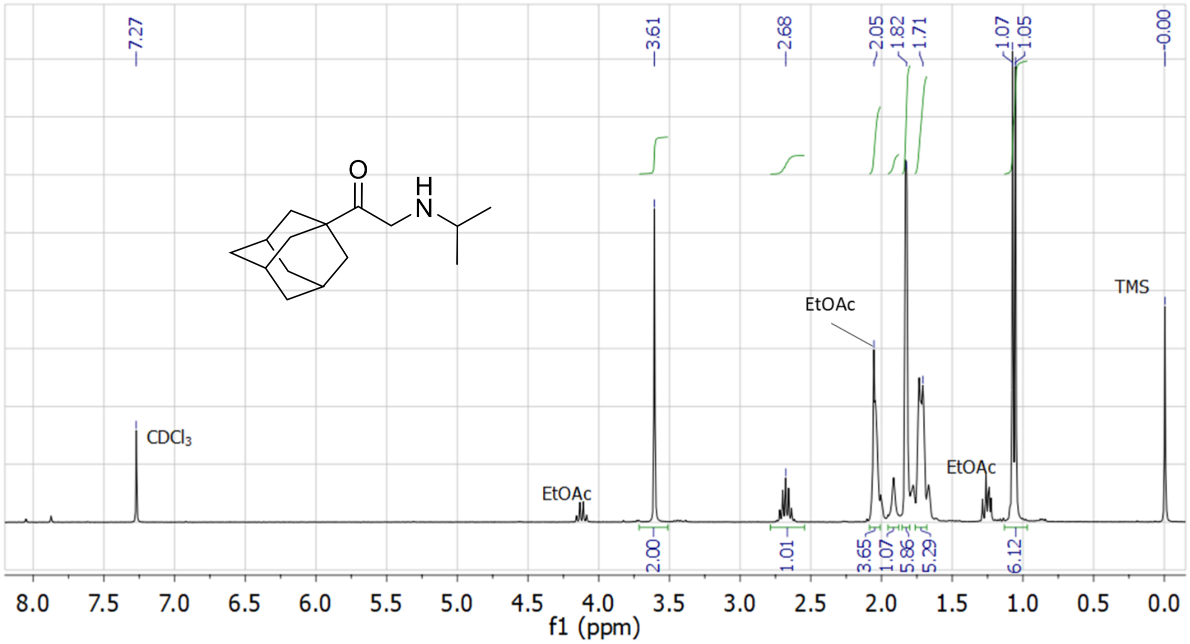

Figure S3. ^{1}H NMR of 1-(adamantan-1-yl)-2-(isopropylamino)ethanone (**2**). ($CDCl_3$, 500 MHz) Peak at δ = 2.08 ppm from residual EtOAc overlaps with product peak at δ 2.05 ppm.

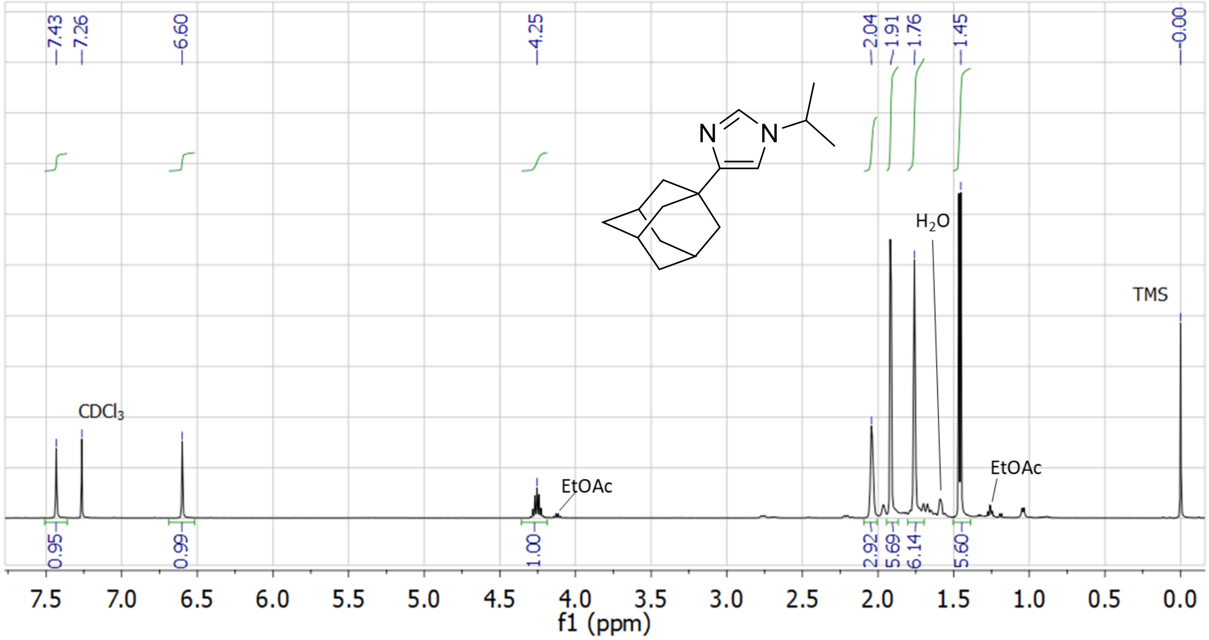

Figure S4. ^{1}H NMR of 4-(adamantan-1-yl)-1-isopropylimidazole (**3**). ($CDCl_3$, 500 MHz).

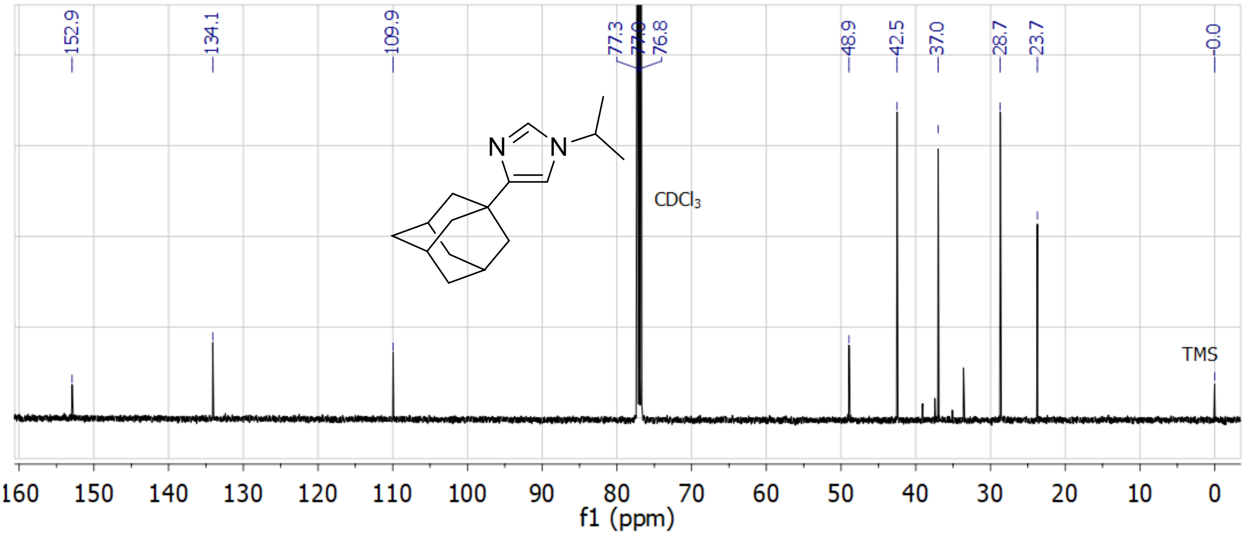

Figure S5. $^{13}C\{^1H\}$ NMR of 4-(adamantan-1-yl)-1-isopropylimidazole (**3**). ($CDCl_3$, 126 MHz). The identity of impurity peaks around δ = 34 to 39 ppm is currently unknown. However, the presence of minor impurities at this stage did not prevent us from being able to access subsequent products **4** and **5**.

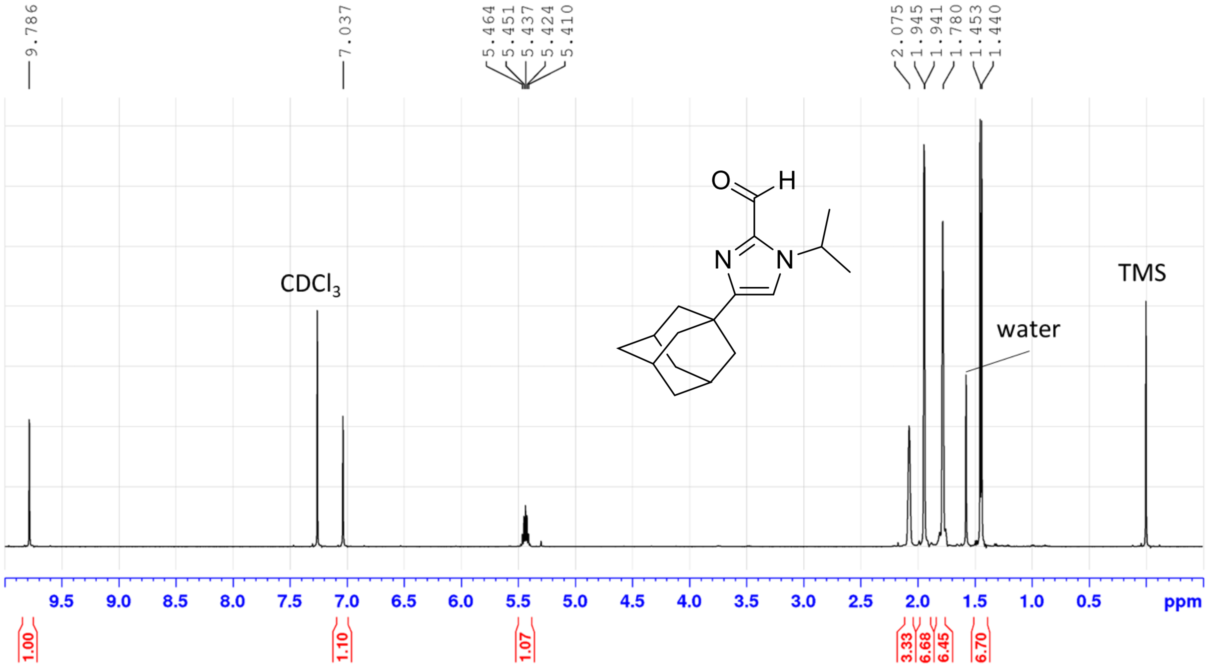

Figure S6. ^1H NMR of 4-(adamantan-1-yl)-1-isopropylimidazole-2-carbaldehyde (**4**). ($CDCl_3$, 500 MHz).

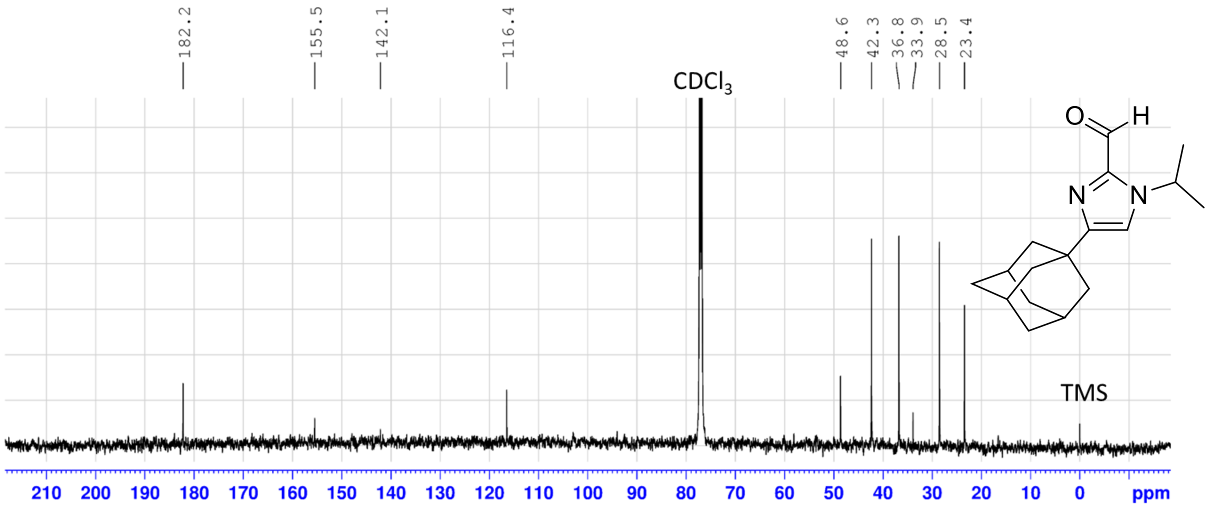

Figure S7. $^{13}C\{^1H\}$ NMR of 4-(adamantan-1-yl)-1-isopropylimidazole-2-carbaldehyde (**4**). ($CDCl_3$, 126 MHz).

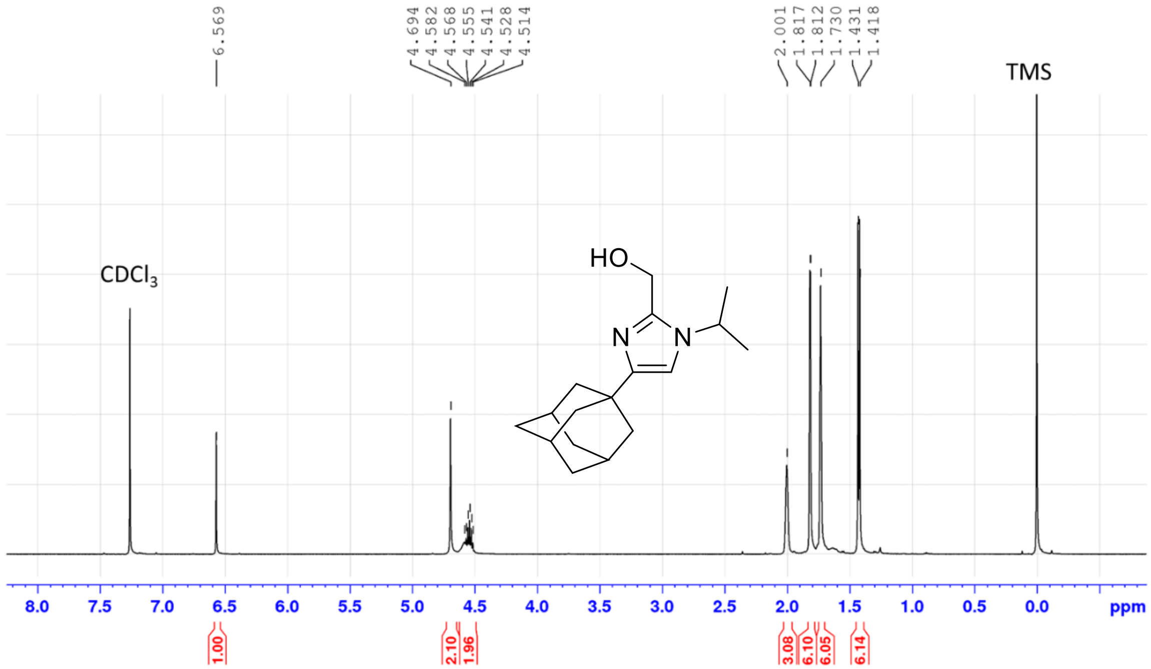

Figure S8. ^1H NMR of (4-(adamantan-1-yl)-1-isopropylimidazol-2-yl)methanol (**5**). ($CDCl_3$, 500 MHz).

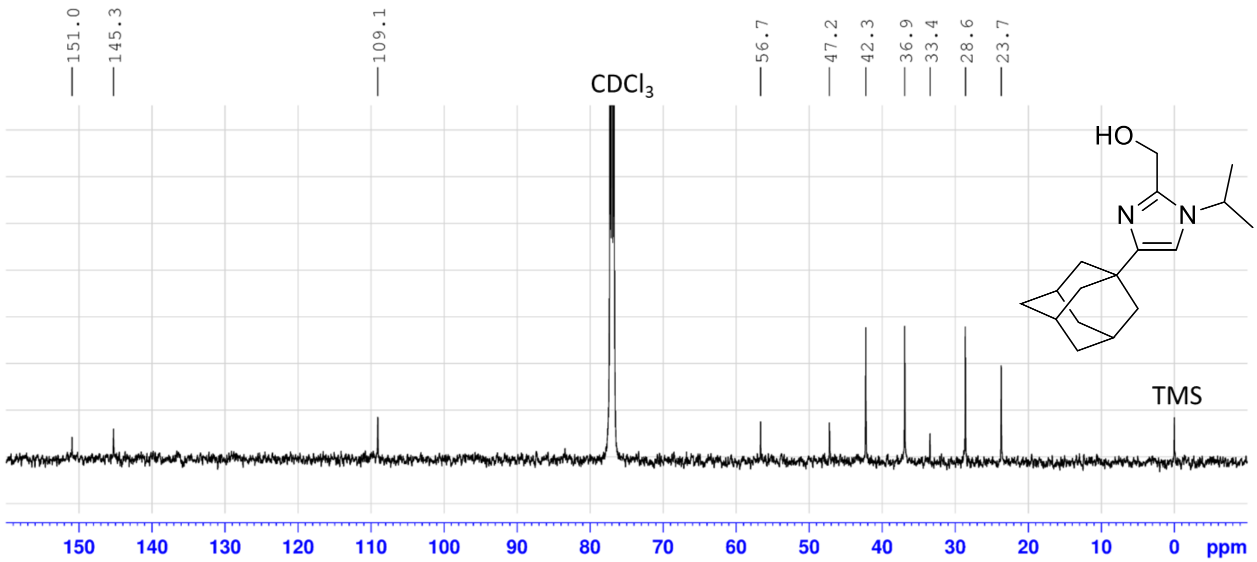

Figure S9. $^{13}C\{^{1}H\}$ NMR of (4-(adamantan-1-yl)-1-isopropylimidazol-2-yl)methanol (**5**). ($CDCl_3$, 126 MHz).
